# Design and Fabrication of Broadband InGaAs Detectors Integrated with Nanostructures

**DOI:** 10.3390/s23146556

**Published:** 2023-07-20

**Authors:** Bo Yang, Yizhen Yu, Guixue Zhang, Xiumei Shao, Xue Li

**Affiliations:** 1 State Key Laboratories of Transducer Technology, Shanghai Institute of Technical Physics, Chinese Academy of Sciences, Shanghai 200083, China; bobyang@mail.sitp.ac.cn (B.Y.); yuyizhen@mail.sitp.ac.cn (Y.Y.); 15216703892@163.com (G.Z.); 2 Key Laboratory of Infrared Imaging Materials and Detectors, Shanghai Institute of Technical Physics, Chinese Academy of Sciences, Shanghai 200083, China

**Keywords:** InGaAs detector, visible-extended, nanostructure, antireflection, Mie

## Abstract

A visible–extended shortwave infrared indium gallium arsenide (InGaAs) focal plane array (FPA) detector is the ideal choice for reducing the size, weight and power (SWaP) of infrared imaging systems, especially in low-light night vision and other fields that require simultaneous visible and near-infrared light detection. However, the lower quantum efficiency in the visible band has limited the extensive application of the visible–extended InGaAs FPA. Recently, a novel optical metasurface has been considered a solution for a high-performance semiconductor photoelectric device due to its highly controllable property of electromagnetic wave manipulation. Broadband Mie resonator arrays, such as nanocones and nanopillars designed with FDTD methods, were integrated on a back-illuminated InGaAs FPA as an AR metasurface. The visible–extended InGaAs detector was fabricated using substrate removal technology. The nanostructures integrated into the Vis-SWIR InGaAs detectors could realize a 10–20% enhanced quantum efficiency and an 18.8% higher FPA response throughout the wavelength range of 500–1700 nm. Compared with the traditional AR coating, nanostructure integration has advantages, such as broadband high responsivity and omnidirection antireflection, as a promising route for future Vis-SWIR InGaAs detectors with higher image quality.

## 1. Introduction

In the shortwave infrared band, many substances have unique spectral characteristics, which can provide low-cost, highly reliable and practical imaging technology support for spectral analysis and infrared imaging. Compared with other shortwave infrared detectors, the infrared indium gallium arsenide (InGaAs) detector has the advantages of high detectivity, high uniformity, high stability and good radiation resistance at near room temperature [1], so it is one of the best choices for miniaturized, low-cost and high-reliability shortwave infrared detection systems and has been widely used in the fields of space remote sensing, astronomical observation and spectral imaging [2,3,4,5].

In order to avoid the performance attenuation of the detector near room temperature caused by lattice mismatch, the conventional InGaAs material has an In component of 0.53, at which the InGaAs material is completely lattice-matched with the InP substrate material, and the corresponding band gap is 0.75 eV, which results in a cut-off response wavelength of 1.7 μm. Due to strong absorption by the InP cap layer (for front-side illumination) or InP substrate (for back-side illumination), the InGaAs detector has a front response cut-off wavelength of 0.9 μm.

For the application of low-light night vision, the energy of incident light, including atmospheric glow, moonlight, starlight, etc., is concentrated in the 0.4~1.7 μm band. The current mainstream low-light night vision devices are mainly equipped with super second-generation and third-generation image intensifiers or Si-based devices [6], which show high sensitivity in the visible band, but the spectrum in the shortwave infrared band is not well matched with the energy distribution of atmospheric glow [7], resulting in limitations for detection on a moonless night. In addition, in areas such as reconnaissance and agricultural detection, it is necessary to detect both shortwave infrared and visible light simultaneously. Conventional detection systems require two separate detectors (Si and InGaAs) working independently, resulting in complex, weighty and costly systems. In order to overcome this difficulty, Ettenberg et al. proposed a substrate removal method to extend the response of the InGaAs detector to visible light and widened the response band to 400~1700 nm [8]. As the InGaAs detector with a visible-SWIR wide-band response simplifies imaging systems and reduces their size and weight [9,10], it is expected to be ideal for the next generation of low-light imaging equipment and has been widely developed and applied. Based on substrate removal technology, Goodrich, Indigo, Raytheon, Sony and other companies have successively realized wide-band InGaAs detectors [11,12,13], and the typical quantum efficiency reported is 15%~500 nm, 70%~850 nm and 85%~1310 nm [14,15]. Although the response spectrum of the wide-band InGaAs detector is in good agreement with the spectral range of low-light night vision [6], its quantum efficiency in the visible band is still low compared with that of nearly 80% in the shortwave infrared band. Therefore, improving the response of the visible band is an urgent difficulty to be solved.

There are two reasons for the low quantum efficiency in the visible band. The first one is that the InP material has a high absorption coefficient in the visible band, resulting in the useless absorption of incident light below 900 nm. The second one is that the InP surface has high refractive indexes, resulting in signal attenuation in the wide-band range. For the first problem, the structure of the contact layer should be effectively optimized, for example, by optimizing the process to further thin the contact layer or using different contact layer materials, such as InAlAs, to suppress the loss absorption. For the second problem, an antireflection (AR) coating should be applied to suppress the surface reflection in the whole visible–shortwave infrared (Vis-SWIR) band, while the fabrication process should be compatible for the back-illuminated infrared focal plane array (FPA).

For InGaAs detectors, the most mature and low-cost AR coating is one or several layers of dielectric film. Rouvié et al. used SiO_2_/TiO_2_ to enhance the infrared absorption of InGaAs layers, which increased the quantum efficiency of the device to about 75% [16]. Kolodeznyi et al. studied the effect of SiNx with different film thicknesses on the reflectivity of InGaAs/InP PIN photodetectors and found that when the SiNx thickness was 200 nm, the reflectivity between 1300 and 1550 nm of the device could be reduced to less than 10% [17]. Traditional dielectric films have important application value for infrared detectors, considering their capability of scalable batch production. However, the intrinsic refractive index of common dielectric materials and the difficulty of accurately controlling their thickness are barriers for the broadband and omnidirection AR coating on InGaAs FPAs. Also, the introduction of extra stress from the multilayer AR coating may harm the long-term stability of Vis-SWIR InGaAs FPAs, as the total thickness of PIN arrays is lower than 4 μm.

In recent years, a novel optical metasurface has been considered a solution for high-performance semiconductor photoelectric devices as it allows the electromagnetic field to be manipulated. Among these optical metasurfaces, the periodically arranged subwavelength structure arrays have been favored due to their compatibility with the State-of-the-Art IC fabrication process. For example, periodical metal plasmonic resonators have been widely used in photovoltaic devices [18], biosensors [19], metamaterials [20] and other fields. However, metal plasma can dissipate a large amount of incident light energy, which, in turn, decreases the conversion efficiency of photoelectric devices, especially image sensors like InGaAs FPAs. The scattering characteristics of metal plasma nanostructures are usually only controlled by electrical resonance, which is not conducive to the regulation of optical fields [21,22]. In contrast, the nanostructure of all-dielectric materials can not only reduce the dissipation loss but also excite electrical resonance and magnetic resonance at the same time, thereby generating a huge resonance antenna in the near field. Therefore, manipulating light-induced resonance in the subwavelength structure of all-dielectric materials (ADMs) is a new hot spot in the field of nanophotonics [23].

In this paper, recent research on the design and fabrication of broadband InGaAs detectors is demonstrated. Based on the fabrication of Vis-SWIR InGaAs detectors using a thin InP buffer layer material, the different methods to improve the response of the visible band were studied. The research on the precise control technology for an ultrathin InP contact layer was carried out, and a high quantum efficiency in a wide spectrum range was realized. And then, the growth and verification of a double-layer antireflection film was realized based on the results of a simulation, and the surface reflectivity and quantum efficiency of the detector after penetration enhancement were tested. At the same time, on the grounds of the antireflection properties of the ADMs, the design and preparation of InP nanoparticles were explored, and the performances of visible–extended InGaAs detectors integrated with the nanostructures were investigated. Finally, the development of an InGaAs detector with a high response throughout the visible and shortwave infrared band was realized.

## 2. Materials and Methods

### 2.1. Fabrication of the Broadband InGaAs Detector

The epitaxial material used in this paper was an NIN structure InP/InGaAs/InP double hetero-junction material, which consisted of a 1 μm thick n-type InP cap layer, a 2.5 μm thick lightly doped In_0.53_Ga_0.47_As absorption layer and a heavily doped n-type InP contact layer. The material was grown using molecular beam epitaxy (MBE), with a 200 nm thick In_0.53_Ga_0.47_As etching barrier layer inserted between the InP buffer layer and the substrate to protect the buffer layer from wet etching during the substrate removal process. A planar structure was chosen for the photosensitive chip, and the p-n junction was formed using Zn impurity diffusion process. The p-n junction was buried in the absorption layer, which reduced the influence of the interface and avoided the difficulty of surface passivation, so the device showed relatively low dark current and noise. A SiN_x_ film was deposited as a diffusion mask using plasma-enhanced chemical vapor deposition (PECVD), and the diffusion hole was etched using inductively coupled plasma (ICP) etching, and the N electrode hole was opened by combining wet etching and ion beam etching. After that, another SiN_x_ film was deposited as a passivation layer, and then P and N Au electrodes were grown using ion beam sputtering. Finally, indium bumps were grown for the electrical interconnection.

**Figure 1:**
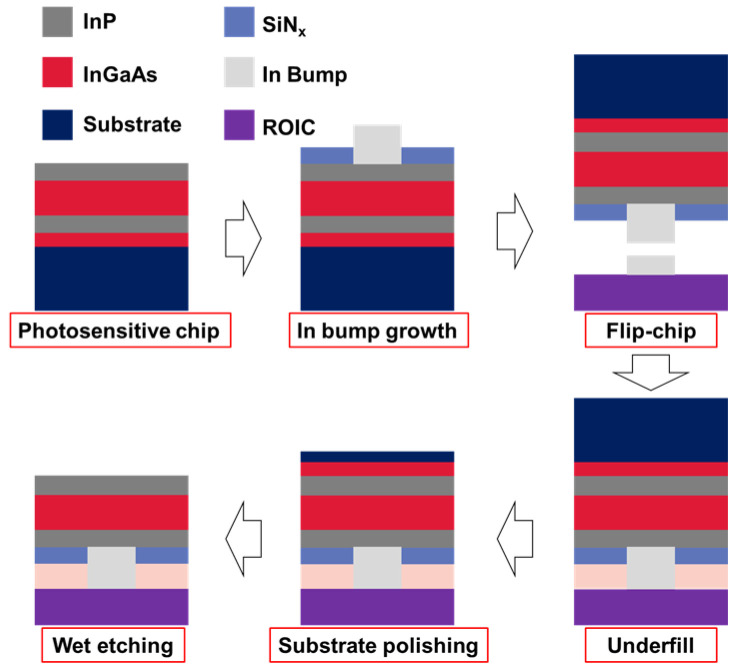
The fabrication flow of the broadband InGaAs detector.

After the chip was prepared, the FPA detector was fabricated using a flip chip technology, in which the photosensitive chip and the readout integrated circuit (ROIC) were bonded with indium bumps for each pixel. And then, the gap between the indium bumps was filled with epoxy resin, and finally, the visible–extended InGaAs detector was achieved by combining chemical mechanical polishing (CMP) and wet etching (Figure 1).

The InP substrate was first thinned to tens of microns using CMP and then was completely removed using wet etching with a solution of a mixture of hydrochloric acid and phosphoric acid with a volume ratio of 3:1 at room temperature. The InGaAs etching barrier layer was then wet etched in a solution of a mixture of tartaric acid and hydrogen peroxide (5:1) at 35 °C temperature. The removal process was monitored by observing the change in the flatness under an optical microscope. Due to undercut problems during the wet etching process and the several-micrometer thickness of the chip, any defect in the material could result in a large area of blind elements.

### 2.2. Ultrathin InP Buffer Layer Controlling Technology

In order to further restrict the absorption of incident light in the visible band, the thickness of the InP buffer layer needs to be further reduced. One way is to grow an ultrathin InP buffer layer during the epitaxy of the material, but it is easy to cause damage in the substrate removal process. The other way is to develop a way to additionally thin the buffer layer after the FPA is prepared. In order to avoid undercut introduced by wet etching, the dry etching process was adopted to achieve ultrathin buffer layer.

The dry etching technique used in this experiment was ICP etching technology. The etching gas was mixture of Cl_2_, CH_4_ and H_2_, and the RF power was set to 80 W. The results with ICP power set to 1000 W, 800 W, 600 W and 400 W, respectively, were compared, and it was found that the etching rate decreased by lowering the ICP power, which was for the benefit of accurately controlling the etching thickness, but, at the same time, etching residues appeared, which led to performance degradation. Therefore, optimization of the process parameters were then balanced between the etching rate and the surface state.

### 2.3. Traditional Antireflection Film Deposition

Referring to the spectral response range of the broad spectrum InGaAs detector (400–1700 nm), research on traditional antireflection film deposited on InP substrate was carried out.

Considering that the transparent window of ZnS covered 0.38–14 μm and that of SiO_2_ could extend into the ultraviolet band, these two materials were selected for the antireflection film. The structures of the ZnS/SiO_2_ film were designed and optimized using TFCalc film design software based on the stacking formula (Table 1). The results show that the average reflectivity from 400 nm to 1700 nm of InP substrate could be reduced to 7.0% and 3.3% by ZnS/SiO_2_ bilayer film and ZnS/SiO_2_ four-layer film, respectively.

Based on the simulation and design results, a single-layer dielectric film and a double-layer dielectric film were grown on the surface of the broad spectrum InGaAs detector module.

### 2.4. Antireflection Nanostructure Integration

The surface microstructure can reduce reflection of incident light according to Mie scattering theory. When the size of the microstructure is much larger than the wavelength of the incident light, the incident light will be reflected multiple times on the surface, increasing the optical path, thereby reducing the reflection. And when the surface microstructure size is smaller than the wavelength of the incident light, the refractive index of the microstructure can be understood as gradually changing, and the material with continuous refractive index of incident light can also achieve the purpose of reducing reflection.

**Figure 2:**
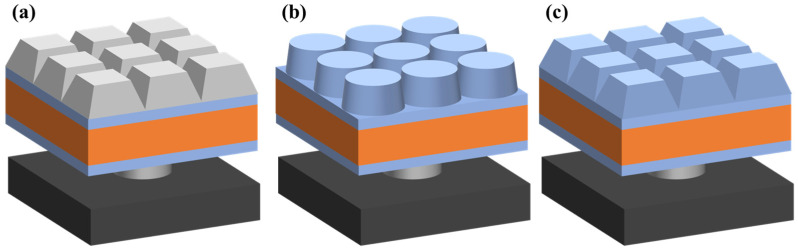
Schematic of textured InGaAs focal plane arrays: (**a**) SiNx nanocone, (**b**) InP nanopillar, (**c**) InP nanocone.

SiNx is one of the commonly used antireflection materials and was chosen for fabrication of Mie scattering AR nanostructure (Figure 2a). The SiNx gradient nanocone was designed and analyzed and was equivalent to a continuous thin film structure with a uniform distribution of multiple layers of refractive index, i.e., equivalent media theory (EMT). And meanwhile, the InGaAs detector consisted of a 350 μm InP substrate; therefore, as a high refractive index material, the direct selection of InP substrate as the material for subwavelength AR nanostructure provided convenience for experimental preparation. Two InGaAs detector device structure models with integrated InP nanostructures on the surface were designed, including the InP nanopillar array (Figure 2b) and the InP nanocone array (Figure 2c).

**Figure 3:**
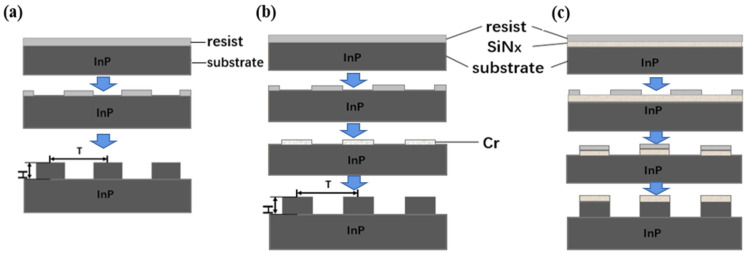
A flowchart of the fabrication process for InP nanostructure arrays with three different masks: (**a**) photoresist, (**b**) metal, (**c**) silicon nitride.

As electron beam lithography (EBL) technology has the advantage of easy adjustment of structural parameters in the experimental stage, it was used for process exploration. During the experiment, three mask materials were explored, including photoresist (Figure 3a), metal (Figure 3b) and silicon nitride (Figure 3c), for the preparation process of InP nanostructure arrays. The nanostructure was realized using ICP etching with the gas mixture of Cl_2_, CH_4_ and H_2_.

Based on the FDTD analytical method and preparation process exploration, InP nanopillar arrays were integrated directly on a conventional shortwave infrared InGaAs detector (SWIR IGA detector) as well as a visible–shortwave infrared detector (Vis-SWIR IGA detector) to verify the practical effect of the AR nanostructures. The parameters of the microstructures were optimized to target the specific response band.

## 3. Results

### 3.1. Performances of the Detector with Ultrathin InP Buffer Layer

The reflectivity of the broad spectrum InGaAs detector in different states was tested. The results show that the reflectivity of the InGaAs detector was higher than 30% over the entire operating band after the conventional fabrication and was slightly influenced by the substrate removal process. After ICP etching, the reflectivity of the focal plane decreased significantly, and the average reflectivity was 12.8%. The decrease in reflectivity was due to the fact that the surface of the dry etched InGaAs detector had some irregular deformation and texture, which caused multiple reflections at the interface, leading to a reduction in surface reflectivity [24].

The responsivity of the InGaAs focal plane detector before and after the visible–extended process was measured. For the wavelength of 900–1700 nm, the InGaAs focal plane detector with the substrate had a significantly low response, mainly due to the absorption of the additional InGaAs etching barrier layer of the material. The responsivity of the broadband InGaAs focal plane detector (~10 nm InP) was significantly improved in the entire working band range of visible–shortwave infrared, reaching 0.31 A/W and 0.73 A/W at 600 nm and 1200 nm, respectively, and achieving a high responsivity level of more than 0.24 A/W in the 500–600 nm range.

The optoelectronic performances of the InGaAs detector before and after the process were tested using the same test system, and the average values of the parameters were calculated (Table 2). It could be seen that the blackbody response signal increased by 7.6%, mainly resulting from the improvement in the visible band. The nonuniformity and the blind pixel rate significantly increased due to the defects introduced by the etching process. In addition, a bimodal phenomenon appeared in the distribution of the response signal, which was caused by etching inconsistencies.

### 3.2. Traditional Antireflection Film Deposition

The reflectivity of the InGaAs detector modules was tested, and the average results with the single-layer AR film and the double-layer AR film were 20.54% and 9.21%, respectively, which was 11.5% and 22.83% lower than the average reflectivity of the traditional InGaAs focal plane. Compared with the analog value, the measured reflectance peaks and valleys shifted toward the longer wavelength, and the average test value was larger than the simulated one. The reason was that ZnS and SiO_2_ films were deposited on the different devices, and the surface status as well as the environment changed when the detectors were transferred between the different chambers, resulting in the thickness of the film being difficult to accurately control.

**Figure 4:**
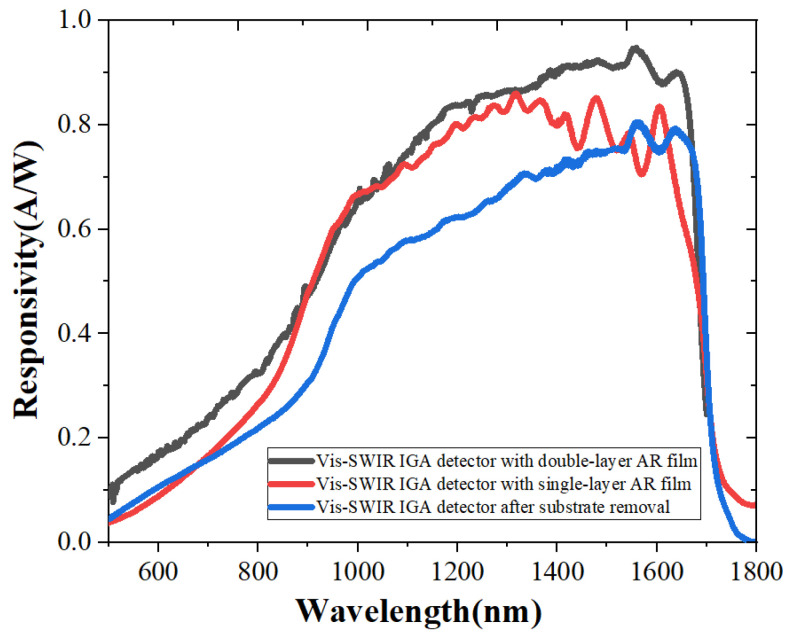
Responsivity of the detectors with and without the AR film.

The response of the InGaAs detector was tested (Figure 4), and the improvement in responsivity under the double-layer AR coating was more obvious. At the wavelength of 800 nm, the responsivity of the detector increased from 0.22 A/W to 0.27 A/W after the single-layer AR film deposition. In comparison, the responsivity increased to 0.33 A/W with the double-layer AR film, and the quantum efficiency of the visible-SWIR InGaAs detector was as high as 24.1% at 500 nm, 35.8% at 600 nm and 75.4% at 1550 nm.

### 3.3. Mie Scattering AR Nanostructure Integration

**Figure 5:**
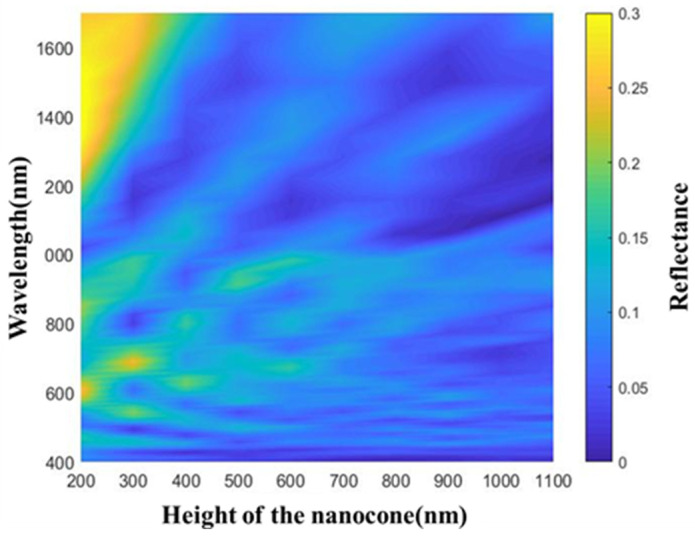
The reflectivity simulation of the nanocone with different heights.

The influence of the microstructure size on the reflectivity of the detector was analyzed with finite difference time domain (FDTD) solutions. Based on the SiN_x_ nanocone simulation model, by changing the period and height and comparing the two structures of a pointed cone and a flat top cone, it was found that the reflectivity was low under the flat top cone simulation model. It can be seen that the reflectivity was lowest at a cone height of 900 nm, when the reflectance curve could almost remain below 10% over the entire operating band range, with an average reflectance of 5.0% (Figure 5).

The reflectivity of InGaAs FPAs integrated with InP nanostructures was also simulated. Both the nanocone array with a diameter of 400 nm and the nanopillar array with a side length of 360 nm were covered with a 150 nm SiNx film. The composite InP nanostructures can maintain a good antireflection effect in the entire working band range, with an average reflectivity of 6.6% for the nanopillar array and 2.4% for the nanocone array.

**Figure 6:**
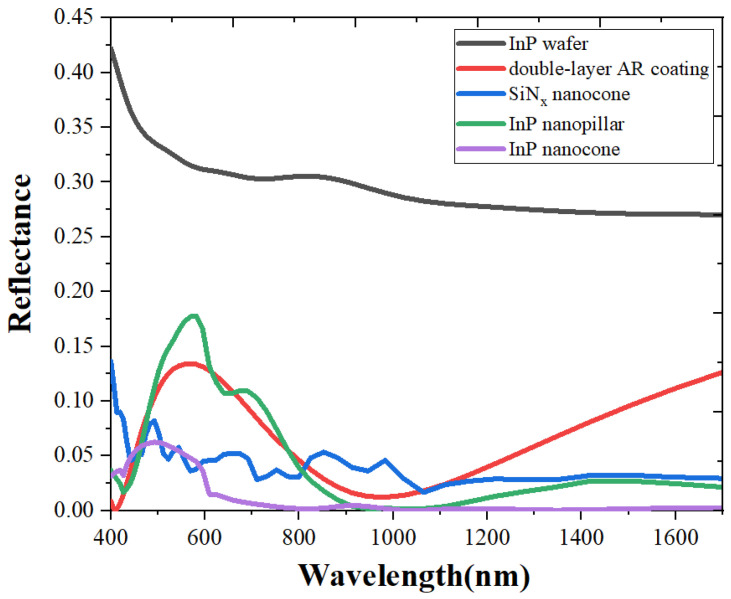
Reflectivity of the InGaAs FPAs with different antireflection systems.

In order to evaluate the AR effect of the composite subwavelength structure, the calculated reflectivity of the conventional InGaAs detector and the traditional double-layer AR film deposited detector was added as a comparison (Figure 6). The InP wafer represents the reflectivity curve of the InGaAs detector without an antireflection design, and the double-layer AR coating stands for films consisting of a 44 nm ZnS layer and a 127 nm SiO_2_ layer. It can be seen that the average reflectivity of the focal plane surface without the antireflection design is 31.9%. The average reflectivity of the double-layer antireflection coating is 7.0%, but the antireflection effect is only better in a narrow-band range.

**Figure 7:**
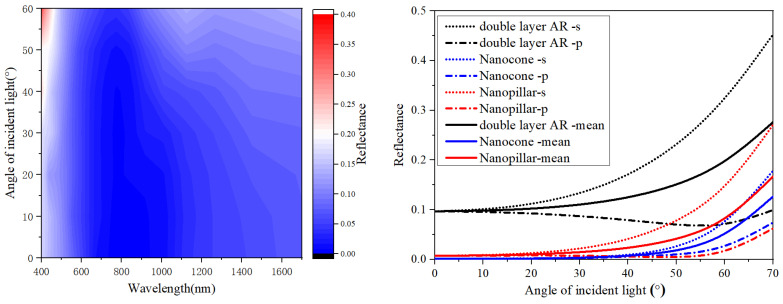
The changes in the reflection of nanocone structure at varied incident angles (**left**) and the comparison of the reflectivity of the different structures vs. angle of incidence at the wavelength of 1550 nm (**right**).

The relationship between the antireflection effect of the different structures and the angle of incident light was analyzed (Figure 7). It shows that the nanocone structure with a changing refractive index echelon can maintain low reflectivity in a wide range of angles and a wide band (Figure 7, left). The reflectivity of the three AR structures with the different angles of S polarized, P polarized and nonpolarized incident light at the wavelength of 1550 nm was compared (Figure 7, right). It can be seen that InGaAs detectors with an integrated nanocone structure on the surface can maintain low reflectivity for light with all different polarized states.

The antireflection performance of the ADM subwavelength nanostructures with wide bands and wide angles mainly comes from two aspects: one is the light-trapping effect of the nanoparticles themselves, and the other is that the subwavelength nanoarrays can be equivalent to a new coating medium, effectively reducing the refractive index jump between the material and the air.

**Figure 8:**
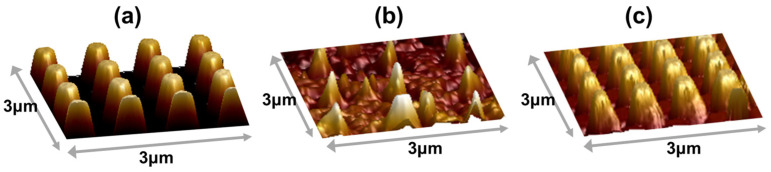
The morphology of InP nanopillars prepared by etching with mask of photoresist (**a**), Cr (**b**) and SiN_x_ (**c**).

The morphology of the InP nanopillars prepared with different etching masks was investigated using AFM (Figure 8). The results show that for the sample etched with the photoresist mask, the top photoresist was etched after the process was completed, and the nanopillars became nanocone-shaped, with poor uniformity (Figure 8a). And for the sample fabricated with the Cr mask, it was found that the verticality of the InP nanopillar was consistent with the design and would not change with the increase in etching time. But the metal mask would reduce the absorption of incident light, so it was necessary to remove the remaining Cr on the surface of the InP nanocolumn. Potassium permanganate was used to remove the Cr mask in this experiment, resulting partly in the corrosion of the InP nanopillars (Figure 8b). In contrast, for the sample etched with the SiN_x_ mask, InP nanopillar arrays with good morphology were obtained after two steps of etching by optimizing the exposure dose (Figure 8c).

After the preparation of the nanostructure on-chip integrated detectors, the photoelectric performances were further tested. The current responsivity of the photosensitive elements of the SWIR IGA detector containing nanostructures reaches 0.43 A/W, 1.06 A/W and 1.17 A/W at 1200 nm, 1500 nm and 1600 nm, respectively, which is 3.6%, 11.2% and 15.0% higher than that of the photosensitive elements without nanostructures.

For the Vis-SWIR IGA detector integrated with nanostructures, 10–20% enhanced the quantum efficiency, and an 18.8% higher FPA response was realized throughout the wavelength range of 500–1700 nm.

## 4. Discussion

**Figure 9:**
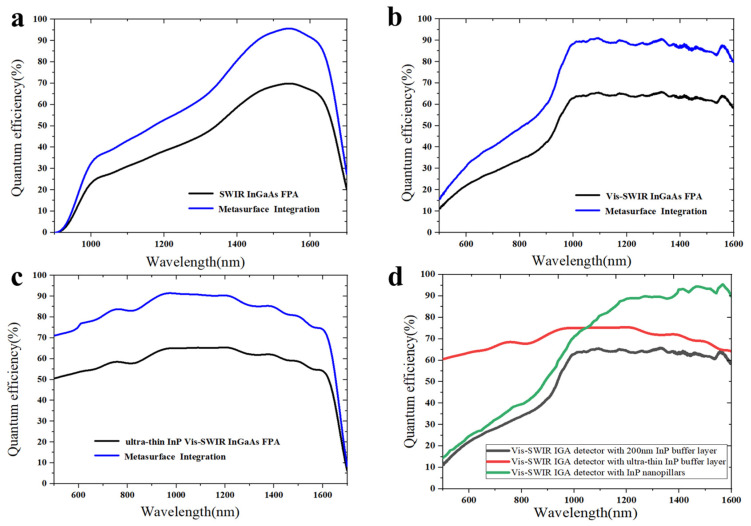
Simulated quantum efficiency of metasurface-integrated (**a**) SWIR InGaAs FPA, (**b**) Vis-SWIR InGaAs FPA, (**c**) ultrathin InP Vis-SWIR InGaAs FPA and (**d**) measured quantum efficiency of the InGaAs FPAs with different systems.

In order to estimate the improvement of the nanostructure on the visible–band response, the quantum efficiencies of the InGaAs detectors integrated with subwavelength nanostructures were compared (Figure 9). In Figure 9a–c, the quantum efficiencies of three types of InGaAs FPAs (black lines) fabricated by our research group were measured. The quantum efficiencies of the metasurface-integrated InGaAs FPA (blue lines) were calculated from the measured FPA based on the FDTD simulation results. By using a well-designed optical metasurface, the response of the InGaAs FPA can be markedly improved. For a typical SWIR InGaAs FPA, which retained a several hundred μm thickness InP substrate, the response property in the region of 1000–1600 nm was entirely raised, especially for the telecom band around the wavelength of 1550 nm. Due to the outstanding antireflection performance, even in Figure 9c, the ultrathin InP vis-SWIR InGaAs FPA was endowed with higher responsivity in the whole spectral region. In Figure 9b,c, the peaks on the spectra curves were caused by the cavity mode resonance, as the PIN arrays on the InGaAs FPA were thinned to ~4 μm. In Figure 9d, the measured quantum efficiency curves exhibited broadband enhancement for the InGaAs FPA in our lab.

The results in Section 3.2 show that the detector with the double-layer coating obtains response enhancement, especially in the visible band (Figure 4). If the number of film layers is continuously increased, a wide-band antireflection film design can theoretically be obtained. The optimization results show that the reflectivity of each point is below 10% in the full spectral range of 400 nm to 1700 nm with the ZnS/SiO_2_ four-layer film system. But the accompanying increased stress of the film layer will introduce additional defects, which will recombine optical carriers and then cause the performance of the device to deteriorate.

As for the ultrathin InP buffer layer, the response is improved over the whole working band, but the number of blind pixels increases obviously because of the substantial occurrence of overheating ones, which is caused by the difficulty of passivating the newly exposed surface of the detector. This creates an inversion layer that traps carriers excited too close to the back surface [18]. At the same time, the difficulties in process control lead to a decrease in the uniformity of the detector response, making it hard to apply to high-quality imaging.

In comparison, the composite InP nanostructures can maintain a good antireflection effect in the entire working band range. Meanwhile, the on-chip integration of InP nanopillar arrays will not introduce the additional noise of the image sensor, and the increase in the full width at the half peak of the response signal is less than 0.01 V. In addition, insensitivity with the angles of incident light and high stability, as well as medium cost, can be realized with the nanostructure integration compared with the other two technical means (Table 3). The results show that the AR nanostructures are the ideal choice for the performance improvement of the Vis-SWIR IGA detectors and are worth further research [25].

In the future, the broad spectrum InGaAs detector described in this paper will be first applied to night vision imaging, and the optimization of the structure design and fabrication process of the different nanostructures will be further carried out. Furthermore, a large amount of the characteristic absorption spectrum information of O-H, C-H, C-O, C=O, N-H and other functional keys is basically located in the 1.7~2.6 μm band. Based on the recent developments in the wavelength-extended InGaAs detector with a response spectrum cover of 1.0~2.6 μm, the design and preparation method of the Vis-SWIR InGaAs detector in this paper has certain reference significance for realizing a broadband InGaAs detector with a spectral operating range of 0.4 to 2.6 μm, which is of great significance for high-quality imaging.

## 5. Conclusions

In summary, we fabricated Vis-SWIR InGaAs detectors using a substrate removal process and then adopted three different technologies to improve their performances, including ultrathin InP buffer layer etching, traditional antireflection film deposition and Mie nanostructure integration. Compared with the traditional film, the AR nanostructures can improve the response of the visible band without a decrease in excellent performance in the shortwave infrared band, realizing a 10–20% enhanced quantum efficiency and an 18.8% higher FPA response throughout the wavelength range of 500–1700 nm, which shows that the nanostructure has good prospects for the fabrication of Vis-SWIR InGaAs detectors with an excellent performance.

## Figures and Tables

**Table 1:** The thickness of each layer and the average reflectivity of the AR film systems.

	ZnS	SiO_2_	ZnS	SiO_2_	Average Reflectivity
Single layer film	-	107.36 nm	-	-	14.7%
Double-layer film	44 nm	127 nm	-	-	7.0%
Four-layer film	67 nm	34 nm	24 nm	118 nm	3.3%

**Table 2:** The optoelectronic performances of the Vis-SWIR InGaAs detector before and after the etching process.

	Blackbody Response Signal (V)	Dark Noise (V)	Dark Signal (V)	Nonuniformity (%)	Blind Pixel Rate (%)
Vis-SWIR detector before etching	1.05	0.0031	0.066	4.2	1.1
Vis-SWIR detector after etching	1.13	0.0028	0.068	12.39	5.6

**Table 3:** The comparison of the performances of the three technical means.

	Response	Reflection (90 Degree)	Reflection (<90 Degree)	Stability	Batch Fabrication	Cost
Ultrathin InP buffer layer	medium	high	high	high	no	medium
Antireflection film deposition	high	low	medium	low	yes	high
Mie scattering AR nanostructure integration	high	low	low	high	yes	medium

## Data Availability

The data that support the findings of this study are available from the corresponding author upon reasonable request.
